# Therapeutic Strategies and Metal-Induced Oxidative Stress: Application of Synchrotron Radiation Microbeam to Amyotrophic Lateral Sclerosis in the Kii Peninsula of Japan

**DOI:** 10.3389/fneur.2022.884439

**Published:** 2022-06-28

**Authors:** Sohei Yoshida

**Affiliations:** Research Center for Neurological Diseases, Kansai University of Health Sciences, Kumatori, Japan

**Keywords:** amyotrophic lateral sclerosis (ALS), gene-environment interaction, parkinsonism-dementia complex, transition metals, synchrotron radiation microbeam

## Abstract

A series of extensive gene-environment studies on amyotrophic lateral sclerosis (ALS) and Parkinsonism–dementia complex (PDC) in Guam Island, USA, and the Kii Peninsula of Japan, including Auyu Jakai, West New Guinea, have led us to hypothesize that a prolonged low calcium (Ca) and magnesium (Mg) intake, especially over generation, may cause oxidative stress to motor and nigral neurons by an increased uptake of environment metallic elements, i.e., aluminum (Al), manganese (Mn), and iron (Fe). Otherwise, 5–10% of total ALS cases are familial ALS (fALS), of which 20% of the fALS cases linked to a point mutation of Cu/Zn superoxide dismutase (SOD1). In the vicinity of the Kii Peninsula, about 7% of the ALS cases are also linked to the SOD1 mutation. Using synchrotron radiation (SR) microbeam, conglomerate inclusion (SOD1 aggregates) within a spinal motor neuron of the fALS case in the vicinity revealed a loss of copper (Cu) in contrast to extremely high contents of Zinc (Zn) and Ca. That means an exceptionally low Cu/Zn ratio with an increased Ca content, indicating the abnormalities of the active site of SOD1 protein of the fALS. Furthermore, sALS in the southernmost high incidence areas of the Kii Peninsula showed a low Cu/Zn ratio within a motor neuron, suggesting a fragility of SOD1 proteins. From the perspective of gene–environment interactions, the above two research trends may show a common oxidative stress underlying the neuronal degenerative process of ALS/PDC in the Kii Peninsula of Japan. Therefore, it is a crucial point for the prospect of therapeutic strategy to clarify a role of transition metals in the oxidative process in both ALS/PDC, including ALS elsewhere in the world. This paper reviews a history of the genetic epidemiological studies, especially from the aspect of gene–environment interaction, on ALS/PDC in the Kii and Guam high incidence foci and the results of a series of analytical research on trace metallic elements within neurons of both sALS and fALS cases, especially using a synchrotron radiation (SR) microbeam of Spring-8 and Photon Factory of Japan. The SR microbeam is an ideal X-ray source, which supplies an extremely high brilliance (high-intensity photon) and tunability (energy variability) to investigate trace metallic elements contained in biological specimens at the cellular level, even more without any damages. This research will provide a valuable information about the mechanism of oxidative stress involved in neuronal cell death in ALS and related neurodegenerative disorders. To elucidate the physicochemical mechanism of the oxidative process in neuronal degeneration, it will shed a new light on the therapeutic strategies for ALS/PDC in near future.

## Introduction

**Figure 1:**
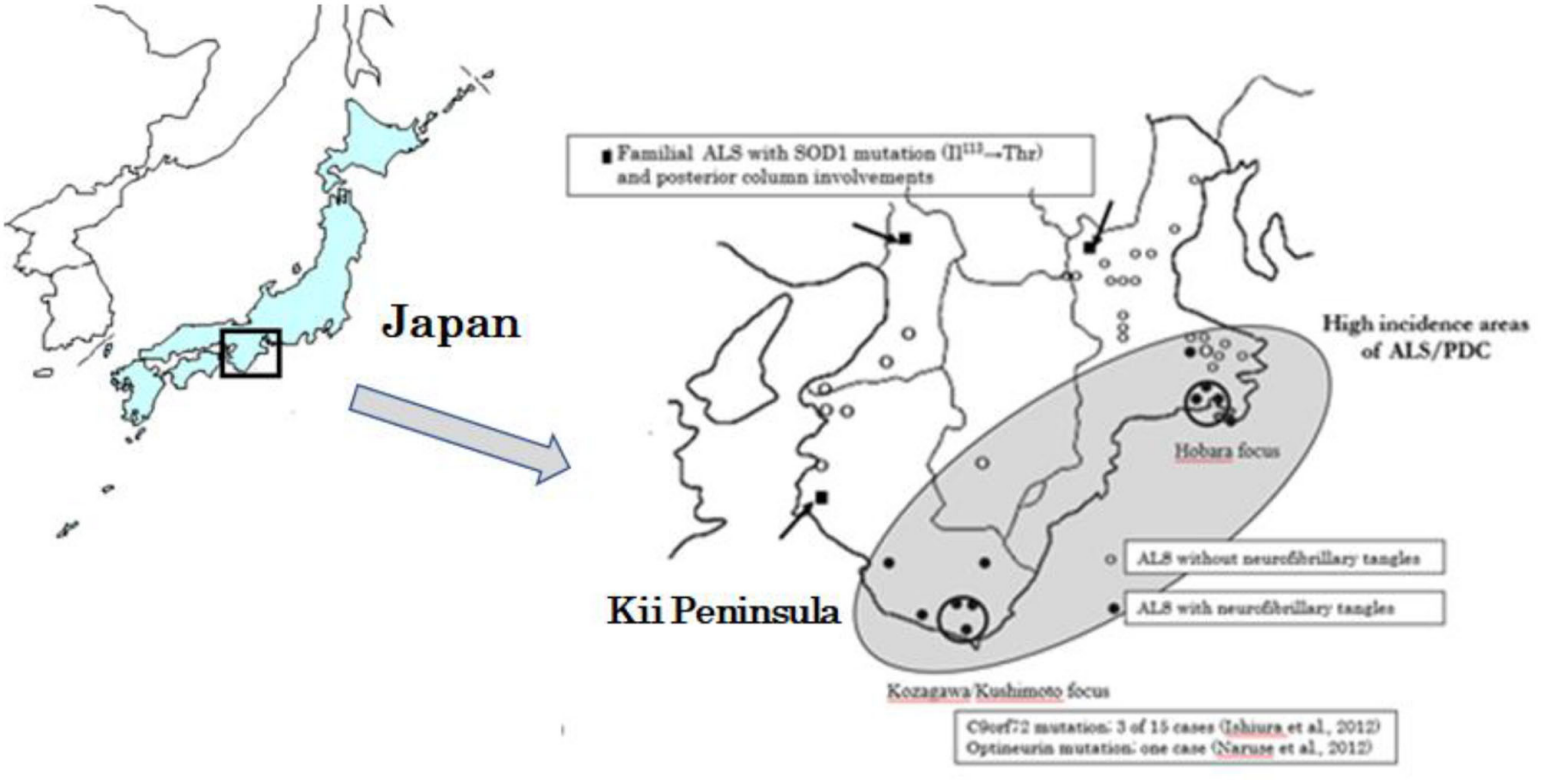
Geographical distribution of ALS cases with neurofibrillary tangles, and with SOD1, C9orf97, and Optineurin gene mutations distributed in the Kii Peninsula of Japan.

Based on the genetic epidemiological studies on Western Pacific foci of Guam, USA, Kii Peninsula of Japan and West New Guinea (Figure 1), Espinosa et al. (1) identified three major forms of ALS: (1) the sporadic or classical form, (2) the familial and dominant genetic form, and (3) the Western Pacific (Mariana Islands) form. The latter form was first described by Hirano (2, 3) among the Indigenous Chamorros people of Guam, often linked to another unique spectrum of disorder—a parkinsonism–dementia complex (PDC). Subsequently, the ALS/PDC was recognized in residents in the two high incidence areas of Kozagawa focus (Kozagawa, Koza, and Kushimoto towns neighboring Kozagawa river, Wakayama Prefecture) and Hohara (Hohara district in Nansei Town, Mie Prefecture) focus in the southern and eastern parts of the Kii Peninsula of Japan (Figure 1) (4) and in the small villages of Auyu and Jakai people of Western New Guinea (5). The excess occurrence of ALS/PDC in these Western Pacific foci had made it the largest and best-known foci of ALS in the world (geographic isolate, initially 50–100 folds of the worldwide average) (6).

To elucidate the causes of the clustering of ALS/PDC, it will provide important clues to understand the causative factors of ALS/PDC. First, this paper presents a brief history of genetic epidemiological studies, especially concerning a gene–environment interaction, on ALS/PDC of the Kii Peninsula and Guam. Second, recent genetic trends of ALS and SOD1 animal models are overviewed. Third, the results of elemental analyses of environmental minerals in the brain and spinal cord tissues of the ALS/PDC autopsy cases and the experimental animal models are presented, using a variety of analytical methods. Specifically, an overly sensitive and high-resolution technique of a synchrotron radiation (SR) microbeam of Spring-8 (Hyogo) and Photon Factory (Tukuba) of Japan was applied at cellular level to elucidate a role of transition metals in the oxidative process of ALS/PDC and related neurodegenerative diseases. Finally, the current trends of the SR microbeam study and the prospect of therapeutic strategy are discussed, especially concerning metal-protein attenuating compounds (MPACs) of transition metals, i.e., Fe, Cu, and Zn.

## Epidemiology and Gene–Environment Interaction

### Genetic Epidemiological Study

A series of genetic epidemiological studies in the Kii and Guam foci since early 1950s (7, 8) revealed that the genetic penetrance did not exceed 20% (9), suggesting a multifactorial inheritance and emphasizing the involvement of environmental factors. Genetics alone could not account for the clusters of ALS/PDC among Chamorros on Guam and other Western Pacific foci as follows: (1) There appears to be three different ethnic groups involved, (2) there appears to be excess of ALS and PDC among Filipinos who settled on Guam as young adults, and (3) there has been a dramatic decline in incidence with a shift of clinical preponderance from ALS to PDC and a significant increase in age at onset of both diseases during the past 30 years (10, 11).

Regarding the environmental factors of ALS/PDC, there were mainly two hypotheses in the Western Pacific foci of the Kii Peninsula and Guam: (1) an imbalance of environmental minerals, resulting in a long-term Ca and Mg deficiencies (4), and (2) addictions to excitatory amino acids (β-methyl-amino-L-alanine; BMAA) in cycads (12–14). However, the residents in the Kii Peninsula have no habit to eat or take cycads (15) Moreover, mass spectrometry did not constantly detect a significant amount of BMAA (β-N-methylamino-L-alanine) in the brain tissues of Kii ALS (16). The hypothesis (2) was, therefore, ruled out in Japan (15, 16). However, Spencer et al. have recently reported a medical use as Kampo medicine (17) and presents a hypothesis of genotoxic chemicals (methylazoxymethanol, MAM) derived from seed of the cycad plant (18). As for hypothesis (1), the long-term Ca and Mg deficiencies lead to the increase in transition metals, such as Al, Fe, and Cu in the cerebral cortex and spinal cord tissues (5, 19, 20). Yase (21, 22) concluded that there was a unique pattern of low content (or lack) of Ca and Mg with a high content of Al and Mn in in the environmental specimens, including soil, water, and plant obtained from the high incidence foci and hypothesized that prolonged exposure to these environments would cause abnormal metabolism detrimental to motor neurons. Depending on the results, it is finally emphasized the synergistic effect of genetic and environmental factors as “gene-environmental interaction.”

Since 1980s, in the Kozagawa focus, clusters of ALS have gradually disappeared, accompanying emigrants from the high incidence areas in the southernmost part of the Kii Peninsula (23). However, emigrant ALS out of the focus areas was still highly prevalent. Among the emigrant ALS cases, the youngest age at emigration apart from the Kozagawa focus was 3–5 years in the very early childhood with a very long incubation time over 70 years (23, 24). However, ALS did not develop in the second generation of the emigrants from the high incidence areas, including the Kozagawa focus. Similarly, in the follow-up study of Chamorros emigrated from Guam to California, the mainland of USA, a high incidence of ALS/PDC was observed in the emigrants who had been raised in Guam during their childhood, but no ALS/PDC was observed in their second generation born in the mainland (25). In contrast, immigrants into both the Kii and Guam foci developed ALS/PDC after a long-term stay in the Kii and Guam foci (23, 26).

### Environmental Animal Models

Due to the unique geological and environmental conditions of the Kii Peninsula, we have tried to create animal experimental models. In an experiment animal model of Japanese macaque monkeys reared for a prolonged period in low Ca/Mg and high Al diets, it neuropathologically showed a small diameter of spinal motor neurons with atrophies of nucleus and nucleolus, eventually leading to the decrease in the cell number (27). In other animal models raised under the same conditions, we clarified the shrinkages of the spinal motor neurons with an increased number of spheroids with anti-PHF antibody-positive staining (28–30). It confirmed the accumulation of neurofilaments by induction of abnormally phosphorylated tau protein (31). Interestingly, Mitani et al. (32) pointed out that only low Mg and high Al diet enhanced most likely to absorb Al into the brain of the experimental animals. Oyanagi et al. (33) proved a loss of nigral dopaminergic neurons in the experimental rats raised by only Mg deficiency with high Al diets, especially over the generation from the fetal stage to 1 year of age.

Additionally in the autopsied ALS cases in both Kii and Guam ALS, it is pointed out the presence of multinucleated cells in the cerebellar cortex (4). In sALS, ectopic neurons were also found within the deep white matter of the spinal cord (34). These findings indicated a congenital migration of neurons in the central nervous tissues of ALS under the gene–environment interaction.

## ALS Genetics and SOD1 Animal Model

Genetically, about 5–10% of cases of ALS are a familial form (fALS), and the remainder is a sporadic form (sALS); they are clinically indistinguishable, but genetically possible to differentiate. Recently, genetically identified subtypes of fALS increased in number. In 1993, missense mutations in Cu, Zn superoxide dismutase (*SOD1*) have first reported and accounted for ~20% of fALS (35, 36). After 2008, there continuously found out *TARDBP* (37, 38) and *FUS/TLS* (39, 40) causative genes. Since 2011, the discovery of *C9orf72* causative gene (41, 42), the most frequent causative gene of fALS in the Europe and the United States, has greatly changed the research a landscape of ALS genetics, as a founder effect.

Over 200 different Cu/ZnSOD (SOD1) gene abnormalities have been found in sALS and fALS (35, 36). Motor neuron degeneration was demonstrated in transgenic mice using mutant Cu/ZnSOD cDNA (43). However, knockout mice with the Cu/ZnSOD (SOD1) gene do not develop the disease (44). Only mice expressing mutant SOD1 show neuronal degeneration, indicating “gain of function” rather than “loss of function” (45). In terms of pathogenesis, the following theories have been proposed: 1) peroxynitrite production, 2) oxidative stress by free Cu, 3) aggregation of mutant SOD1, and 4) pathological glycation of mutant Cu/ZnSOD (SOD1). However, the developments of recent genetic studies of fALS, based on the causative gene analyses, are mainly directed toward 1) disturbance of proteostasis, 2) abnormal RNA metabolism, and 3) axonal pathology and cytoskeletal abnormalities (45, 46).

In the Kii Peninsula of Japan, optineurin and C9orf72 causative genes have been found only in the Kozagawa focus (47, 48). In the vicinity of the Kii Peninsula foci, SOD1 abnormalities have also been reported, as in the other Japanese areas (49). Except for the Kozagawa focus, none of the causative genes have been found in the Hobara focus, including in the other Western Pacific high incidence foci of Guam. In this context, the Kozagawa area of the Kii Peninsula occupies genetically a unique feature even in the Western Pacific foci.

From the border of the Kii Peninsula foci, Yoshimasu et al. (50) reported a first case of fALS neuropathologically examined and showed conglomerate inclusions within a motor neuron and posterior column involvements. There was a SOD1 gene analysis on 23 ALS cases (three FALS and 20 sALS cases) from the Kii Peninsula and its vicinity (Figure 1) (51). The two of the sALS cases, outside of the Kozagawa and Hohara foci, showed a Ile113Thr point mutation of exon4. Kokubo et al. (52) neuropathologically examined one of the two cases and found loss of spinal motor neurons, conglomerate inclusions, and posterior column involvements. Incidentally, the Ile113Thr point mutations were reported from various families with an exceptionally low penetrance. Neuropathological examinations revealed a massive accumulation of neurofilaments in spinal motor neurons, globus pallidus, substantia nigra, nucleus pellucida, and inferior olivary nucleus in large amounts (53, 54).

## Trace Elemental Analysis of KII and Guam ALS/PDC

To examine the effects of trace elements, metal dynamics in the CNS tissues of ALS/PDC were analyzed by a variety of methods: X-ray microanalysis (XMA), particle-induced X-ray emission analysis (PIXE), and electron energy loss spectrometry (EELS).

### XMA and PIXE Analyses

The XMA imagines of the spinal cord tissues obtained from autopsy cases of the Kii ALS showed that trace elements such as Al, Ca, and Mn were found to be deposited alternately along the blood supply of intraspinal arteries (55–57). Specifically, these metallic elements deposited along the anterior spinal arteries alternatively in the left or right side of the anterior horn tissues, finally to motor neurons. It indicated that the deposition of trace elements was a phenomenon occurred under hyperparathyroidism due to the long-term deficiency of Ca and Mg intake.

The PIXE analysis showed that increased Al in the spinal cord and frontal cortex tissues obtained from the Guam and Kii ALS/PDC autopsy cases, together with Ca and transition metals of Fe, Mn, Fe, Ti, and V (58). The Al and Ca contents were significantly negatively correlated with ages at onset, and only Ca content was significantly positively correlated with the duration of the illness. Finally, the X-ray powder diffraction and infra-absorption spectrometry revealed that Ca was finally deposited as Ca-hydroxyapatite in the cerebral cortex and spinal cord of ALS (59). Based on these findings, Yase (60) proposed the hypothesis of metal-induced calcifying degeneration as a pathogenesis of ALS, referring to Selye's theory of calciphylaxis (61).

### EELS Study of Ultrastructural Localization of Aluminum

Furthermore, the ultrastructural distribution of Al within a lumbar motor neuron of ALS was analyzed by electron energy loss spectrometry (EELS) (62). Al was localized in nucleoli, nuclei, and rough endoplasmic reticulum, which are rich in phosphorylated nucleic acid components. In addition, Al was found in a Bunina body, which is a pathognomonic intracellular inclusion of ALS. Multivariate analysis of the spinal cord of ALS revealed that the contents of Al and Fe were significantly associated with the frequencies of early pathological changes of chromatolysis and Bunia bodies of spinal motor neurons (63). Al^3+^ has similar characteristics to Fe^3+^ and acts as a prooxidant, inducing reactive oxygen species (ROS) production. Al is non-redox active metal and Al^3+^ firmly binds to metal-binding amino acids (rich in *His, Tyr*, and *Arg*) and phosphorylated amino acids (64). Thus, Al may preferentially bind to nucleic acids and cause a progressive inhibition of the protein synthesis of rRNA and the transcription or gene modulation of DNA (65).

### Application of Synchrotron Radiation Microbeam to ALS/PDC

**Figure 2:**
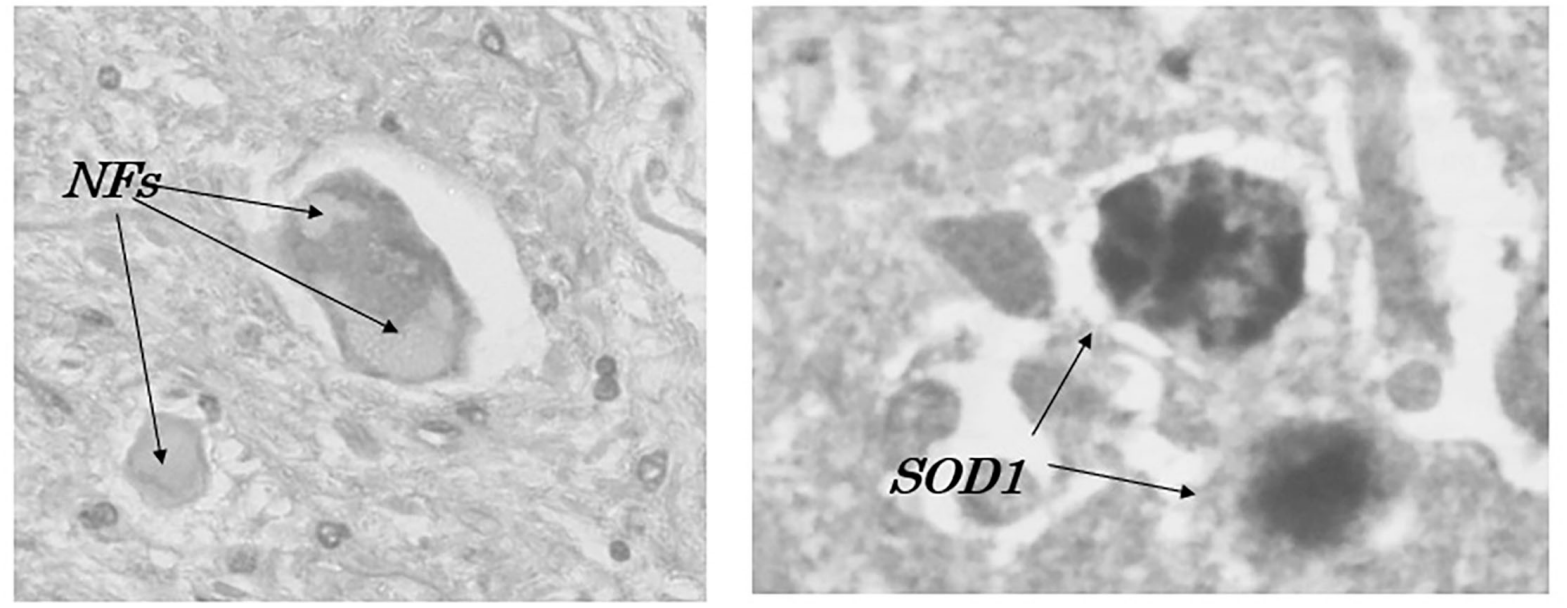
Accumulation of neurofilaments (NFs) and SOD1-immunoreactive products (SOD1) in a patient with familial amyotrophic lateral sclerosis with I113T SOD1 mutation (52).

**Figure 3:**
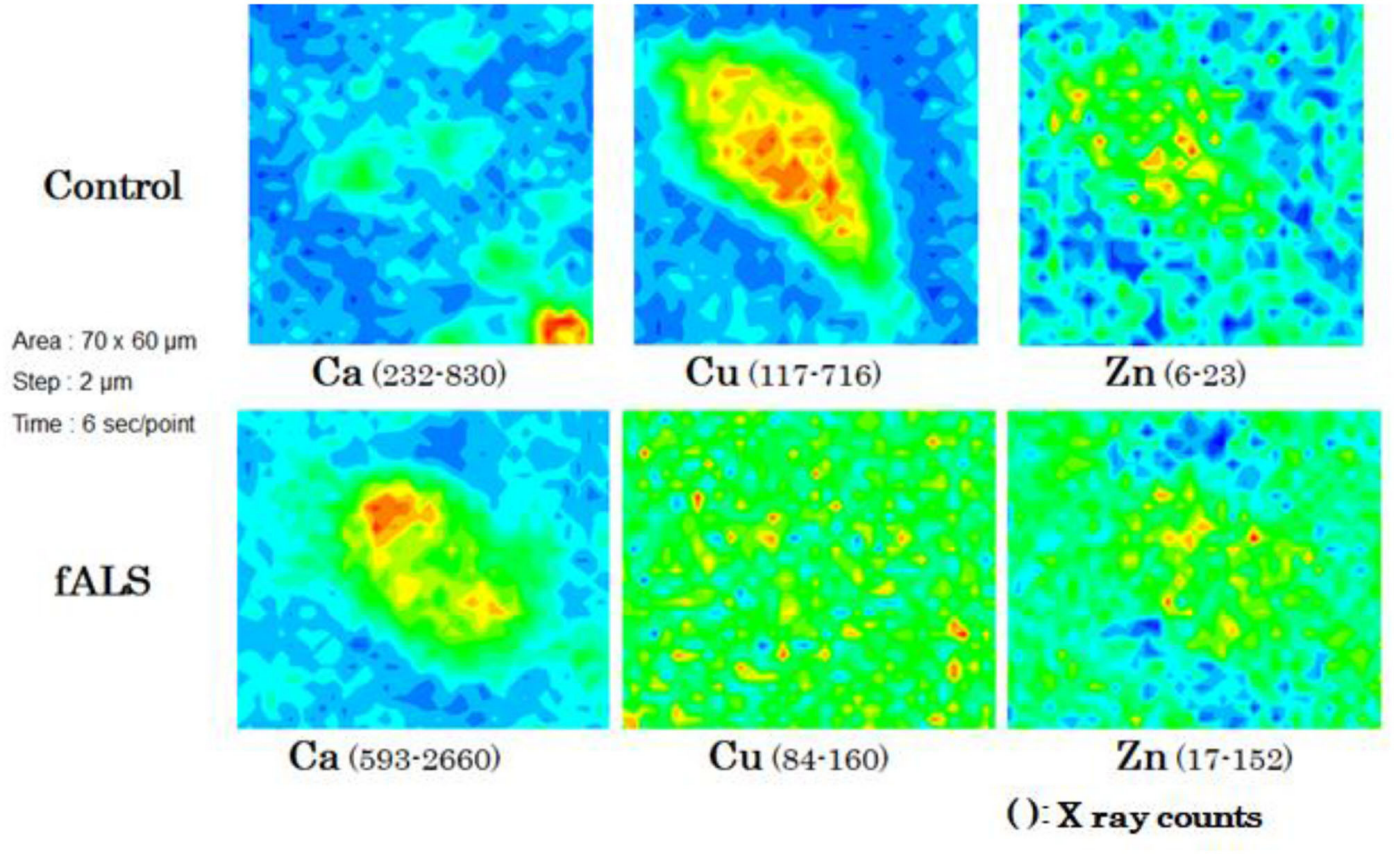
Chemical mappings of Ca, Cu, and Zn of a single motor neuron obtained from fALS and control cases.

To elucidate the physicochemical mechanism of oxidative stress, the chemical states of transition metals such as Cu, Zn, and Fe in the aggregates of mutant Cu/ZnSOD (SOD1) proteins (49, 52) within a spinal motor neuron of autopsy fALS cases were analyzed (Figure 2), using synchrotron radiation (SR) microbeams at Spring-8 and Photon Factory (Figure 3) (66, 67). X-ray fluorescence spectroscopy (SRXRF) using a SR microbeam is non-destructive and extremely sensitive, and its characteristics are suitable for trace elemental analysis within a single neuron and for chemical state analysis of transition metals, providing important information for elucidating the physicochemical properties during the oxidative process (66, 67). Furthermore, SRXRF can image a microdistribution of elements and detect a chemical shift (valency changes) of transition metals in the oxidative process, resulting in cellular death (68).

**Figure 4:**
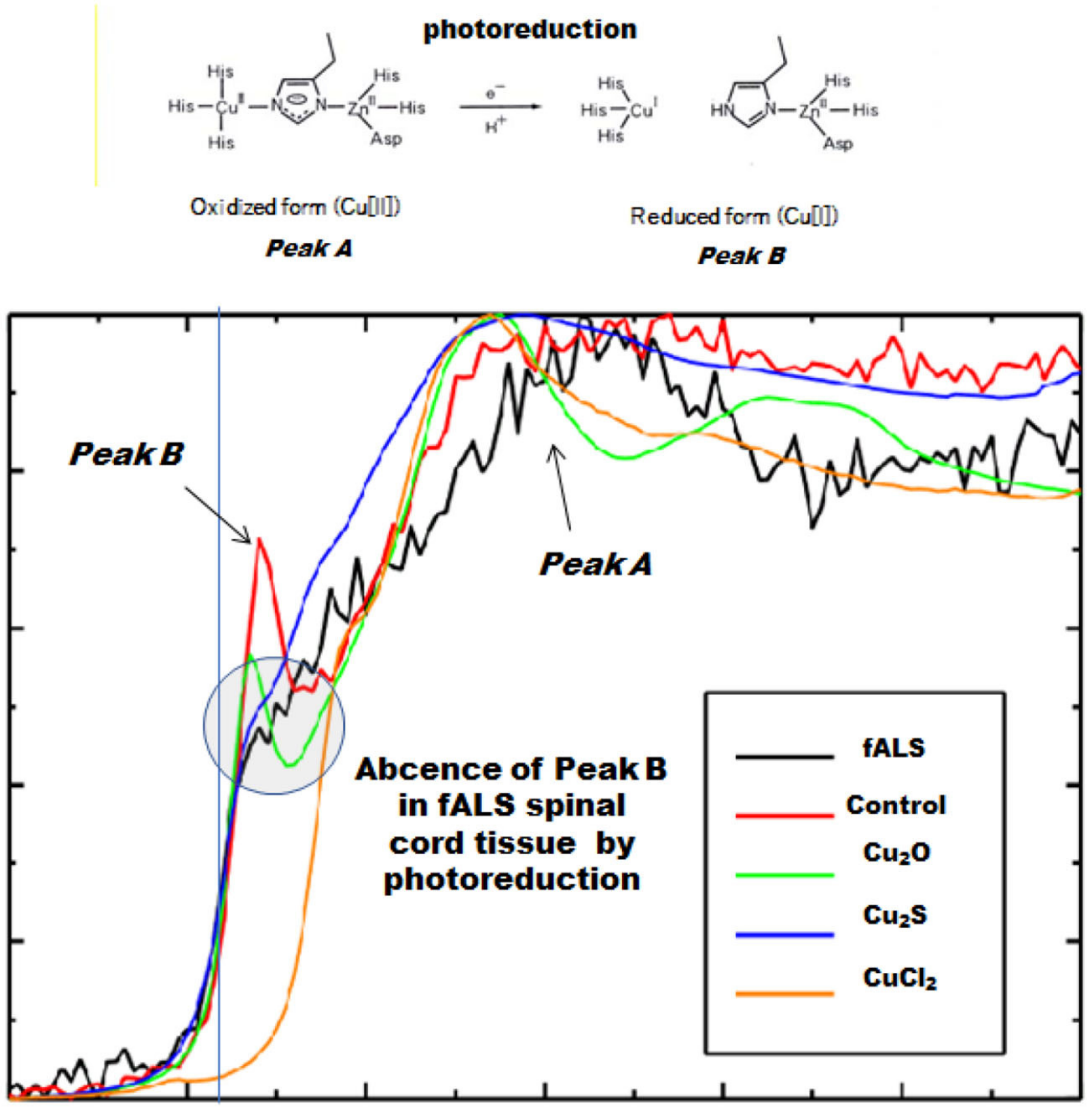
Spectra near the Cu-K absorption edge (XANES). Loss of the pre-edge peak B (~8.983 keV) by photoreduction below the Cu-K absorption edge in fALS-1 but not in control-1.

**Figure 5:**
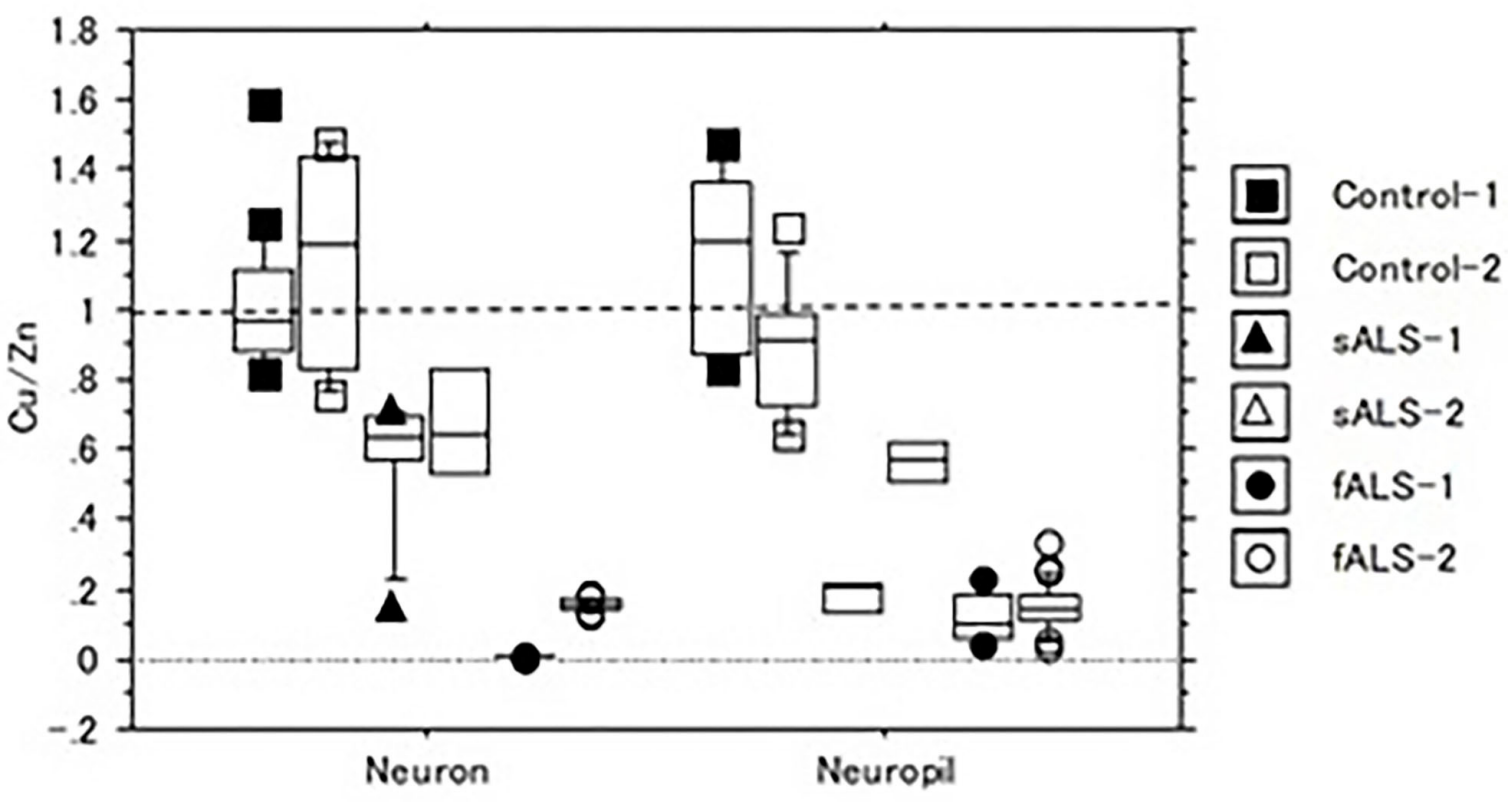
The Cu/Zn ratio of neuron and neuropil in lumbar spinal cord tissues of sALS, fALS and control cases.

**Figure 6:**
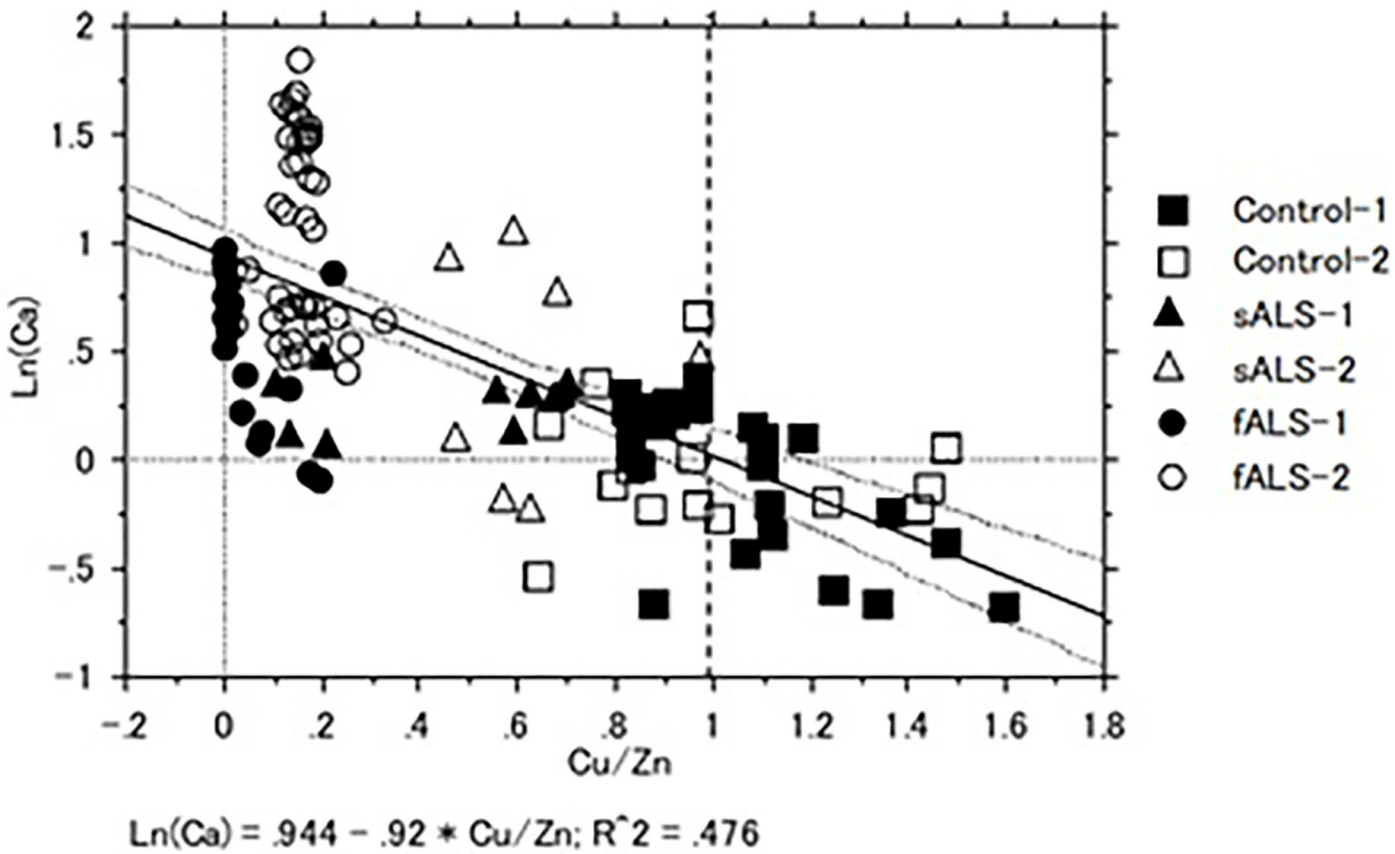
Correlation of Cu/Zn ratios and Ca contents in the lumber spinal cord tissues of sALS, fALS and control cases. Overall, Ln (Ca) = 0.944–0.92*Cu/Zn (*n* = 118, *r* = 0.690, *R*^2^ = 0.476, *p* < 0.0001).

In a case of fALS with a mutant SOD1 protein (Ile113Thr point mutation) (53), the pre-edge peak (~8.983 Kev) below the Cu-K absorption edge was not detected by photo reduction (Figure 4) (66, 67). It indicated the planar triangular structure (Cu-*His*-Zn imidazolate linkage) in the active center of SOD1 protein was impaired before photoreduction (69). The Cu contents in the motor neurons of fALS cases were extremely low, whereas the Zn contents were extremely high, as compared to those of the sALS cases and the controls. Comparing with the Cu/Zn ratios of the control neurons (1.03±0.24), the Cu/Zn ratio of sALS neurons was about half (0.5±0.24) and the Cu/Zn ratio of fALS neurons was extremely low (0.12±0.08). Overall, it was expressed as the equation; ln(Ca)=0.944–0.92^*^Cu/Zn (*n* = 118, *r*= 0.690, *R*^2^ = 0.476, *p* < 0.0001, Figures 5, 6).

**Figure 7:**
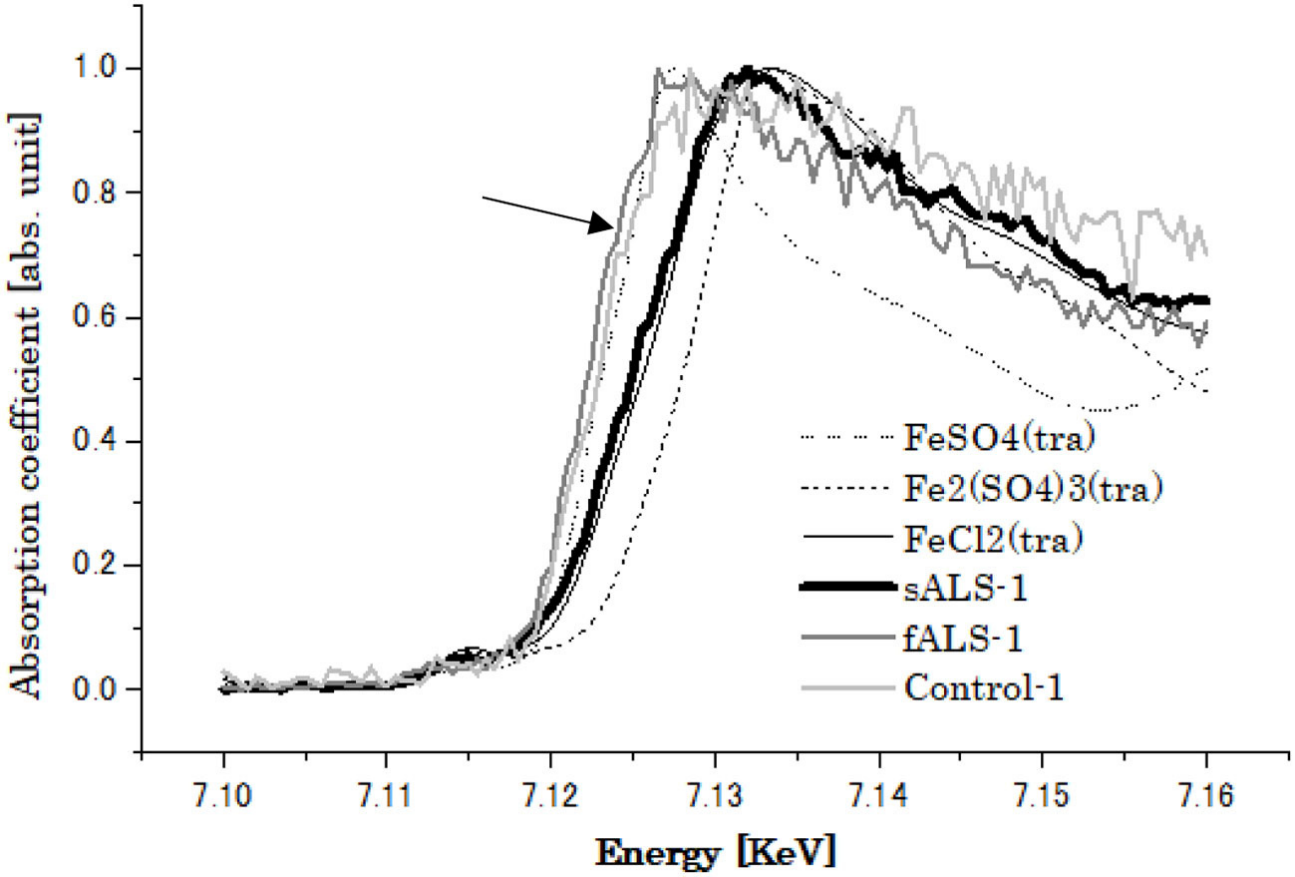
XANES spectra of Fe obtained from of lumbar spinal cords of Control, sALS and fALS cases with SOD1 mutation (indicated by arrow).

On the other hand, the XRF study of one of the sALS cases revealed the highest content of Fe as compared to those of the fALS and control cases. In XAFS study, the chemical state of Fe was found to be shifted from divalent (Fe^2+^) to trivalent (Fe^3+^) as compared to that of the control and fALS cases (Figure 7). The changing of chemical state of Fe might also indicate an important participatory role of Fe in the oxidative process in the sALS case (68, 70), leading to the fragility of SOD1 protein with low Cu/Zn ratio in sALS other than genetic factors.

On the other hand, excess Zn may promote nitration of tyrosine (71) and strongly bound to neurofilament L (NFL) (72), resulting in the neuronal death with the formation of intracytoplasmic neurofibrillary aggregations (conglomerate inclusions). Otherwise, Ca2+ is one of the key regulators of cell survival and it can induce ER stress-mediated apoptosis in various conditions (73). Excess Fe within a motor neuron enhances ER stress and inhibits intracellular Ca^2+^ signaling and ER Ca^2+^ pumps (74), leading to neuronal death.

### Analyses of Iron in Parkinsonism–Dementia Complex and Parkinson's Disease

In Guam, the incidence of ALS has been drastically declined since 1970s and in contrast, that of PDC has gradually decreased, clinically transforming to a mild form of late onset dementia (Mariana dementia) (75, 76). Parkinsonism–dementia complex (PDC), which was previously highly prevalent in the ALS foci of Guam and the Kii Peninsula, was pathologically characterized by extensive Alzheimer's fibrillary changes in the cerebral cortex and brainstem, as well as spinal motor neurons. In PDC, neuronal death with Alzheimer's fibrillary changes is observed in the substantia nigra of the midbrain (2, 3, 77). Here, we compared the role of Fe in the oxidative stress death of nigral neurons in Guam PDC and PD in Japan, using SR microbeam (66–68).

**Figure 8:**
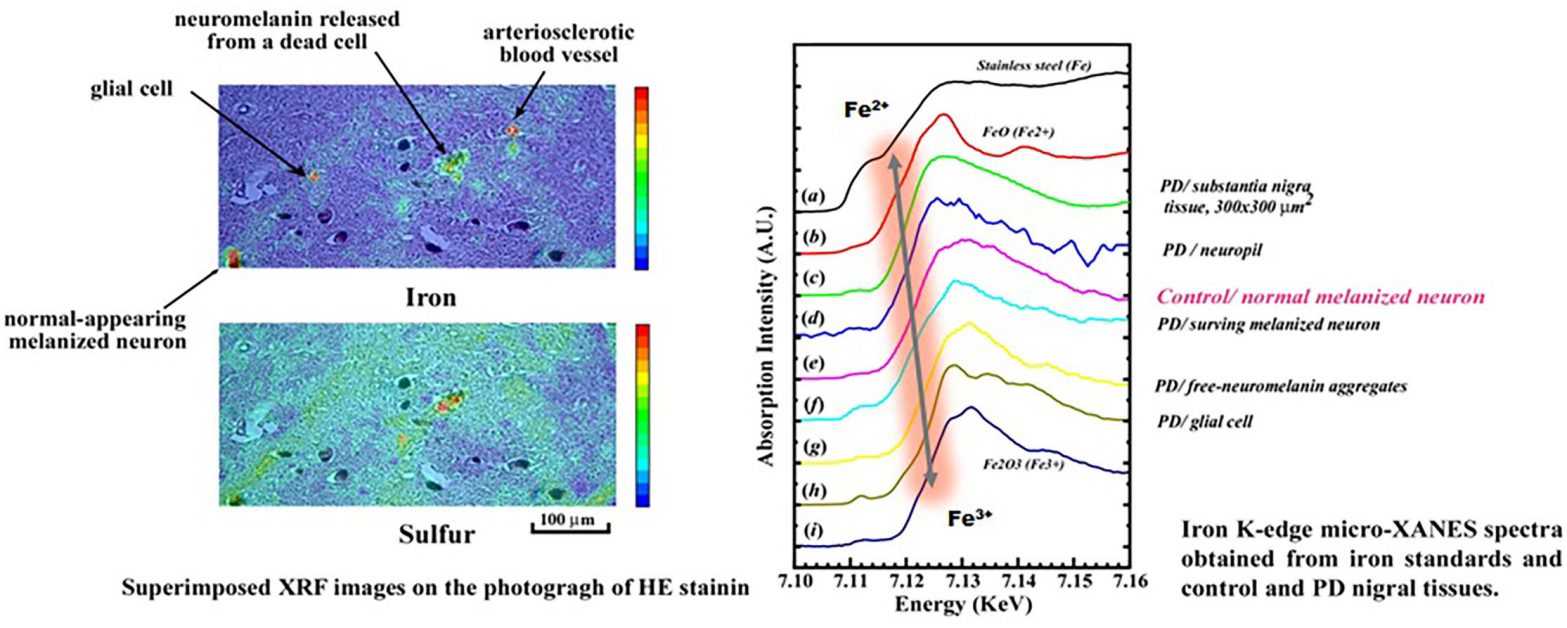
Chemical shifts of Fe from Fe^2+^ to Fe^3+^ in the neuromelanin complex during the Oxidative process of nigral cell degeneration in Parkinson's disease (PD).

To elucidate a role of Fe in oxidative stress underlying the pathogenesis of PDC and PD, distributions of Fe within a nigral neuron were analyzed using synchrotron radiation (SR) microbeam (66–68, 70). In the X-ray fluorescence (XRF) spectroscopic study, excess accumulations of Fe were found within the melanized neurons, free neuromelanin (NM) granules, and NM aggregates phagocytosed in glial cells of the substantia nigra of both PDC and PD. X-ray absorption near-edge structure (XANES) analyses of PD revealed that the chemical state of Fe in the melanized neurons and free NM aggregates or phagocytosed NM aggregates in glial cells shifted from Fe^2+^ to Fe^3+^, according to the progression of oxidative process (Figure 8), associated with a pre-edge peak at Fe K-edge due to a 1s → 3d transition, which indicated a breaking of inversion symmetry around the Fe site (66). However, in the PDC and control, the melanized neurons and free MN aggregates showed mixed states of Fe^2+^ and Fe^3+^ without any pre-edge peak in the spectra. Together with the results, it confirmed that the Fe in the PD nigral neurons was shifted to the trivalent state as “masked iron” (not easily ionized), which does not easily show iron staining properties, and formed a unique tightly bound Fe-NM complex to the membrane proteins (78–80) and not easily ionized. The NM aggregates in PD may be protectively associated with a long-term course of PD. These results suggested that the changes in distribution and chemical states of Fe may play a crucial role in the oxidative process of PDC, but in the different ways other than PD (68).

## New Research Trends and Therapeutic Strategy

### Causative Genes and Oxidative Stress by Transition Metals

Recently, it has been pointed out that oxidative stress caused by reactive oxygen species (ROS) is involved in neuronal cell death in neurodegenerative diseases such as Alzheimer's disease, Parkinson's disease, and amyotrophic lateral sclerosis (81). Intracellular redox reactions involve the transfer of electrons, producing ROS with unpaired electrons. Transition metals, such as iron (Fe) and copper (Cu), have unpaired electrons and change their electronic states relatively easily, generating ROS *via* the Fenton and Haber–Weiss reactions (82), but the detailed mechanism of Fenton's reactions is not fully understood (83). Anyhow, oxidative stress may be deeply involved in the degenerative process of neurons in these neurodegenerative diseases, and the elucidation of such an oxidative mechanism is a crucial point for therapeutic approaches to neurodegenerative diseases.

Using 62Cu-ATSM in positron emission tomography (PET), a significant increase in Fe accumulation in the motor and motor-related cortices has been clinically observed in a group of patients with ALS, and oxidative stress and abnormal mitochondrial function have been pointed out as the pathogenic mechanisms (84). On the other hand, a transactive DNA-binding protein of 43 kDa (TDP-43) has been identified as a major component of intracellular inclusions in spinal motor neurons and ubiquitin-positive inclusions in frontotemporal lobar degeneration with ubiquitinated inclusions (FTLD-U) in sporadic ALS. TDP-43, an RNA-binding protein, has been identified as a major component of the neuronal inclusion bodies in frontotemporal lobar degeneration with ubiquitinated inclusions (FTLD-U), which is recently considered a pathological hallmark in the diagnosis of ALS (85). Interestingly, it is reported that TDP-43 protein is co-localized with Bunina body, which has been considered as a pathognomonic hallmark of ALS (86, 87). In a mouse model with a genetic abnormality of TDP-43 protein (A315T), the aggregation site of TDP-43 protein is enriched in transition metals such as Cu, Zn, and Mn, which may be involved in oxidative stress and neuroinflammation (88, 89). C9orf72 repeat elongation is said to cause mitochondrial dysfunction, oxidative stress, and DNA damage (90). The SR microbeam techniques may provide an important information to elucidate the coordination structure of TDP-43 proteins of ALS-FTD in future.

In addition, it was reported that the cause of the FTD/ALS ancestry linked to chromosome 9 was due to an abnormal expansion of GGGCC repeat sequence in the intron of the C9orf72 gene (41, 90). In Europe and the United States, this repeat extension was found to be the most common cause of familial and sporadic ALS, accompanied by TDP-43 pathology. However, the frequency of the C9or72 repeat expansion is low in Asia. In Japan, it is extremely low, that is, accounting for less than 2.6% of familial ALS and 0.2% of solitary ALS (48, 49, 91) However, the C9orf72 repeat expansion has been energetically investigated in both foci of the Kii Peninsula, and abnormal repeat expansion was frequently observed 20%, three of 15 patients with ALS in the Kozagawa focus (neighboring the Koza river in the Wakayama Prefecture in the southernmost part of the Kii Peninsula) (48). Paradoxically, no C9orf72 repeat expansion has been observed in the other Hohara focus (Nansei Town in the Mie Prefecture), where familial cases are over 70%, as compared to very few in the Kozagawa focus (4). It is supposed to be a founder effect. Even more, in the Kozagawa focus, a ALS case of young onset with long-term survival has been confirmed optineurin gene mutation (47). Therefore, even if gene mutations are involved, causative genes are heterogenous in the Kozagawa focus. The paradox and discrepancy of gene mutations between the Kozagawa and Hohara foci have remained to be elucidated, about 200 Km apart from each other in the Kii Peninsula of Japan.

### Therapeutic Strategy of Metal-Protein Attenuating Compounds

Recently, clioquinol (CQ) has been considered as a moderate chelator/ionophore, only which can across a blood–brain barrier, for the metal-protein attenuating compounds of transition metals, i.e., Fe, Cu, and Zn (92–94). Based on such functions of CQ, researchers began using it for therapeutic purposes in a variety of experimental mouse models and clinical trials, such as Alzheimer's disease (AD) and Parkinson's disease (PD). In transgenic AD mouse models, it was observed that CQ reduced amyloid plaque burden and improved cognitive functions (95). Next, CQ was applied to a human phase II clinical trial for patients with AD (96). The results suggested that CQ prevented cognitive deterioration and reduced plasma Aβ-42 levels. After warning given by the Tabira (97) concerning the SMON tragedy from Japan (2001), the CQ therapy for AD was changed to a derivative of PBT-2 (as a second-generation 8-hydroxyquinoline) (98, 99). However, it was reported that the chelating properties of CQ depleted copper and increased lethality in amyloid precursor protein transgenic mouse (100). On the other hand, it was demonstrated that iron chelation by ferritin transgene or the metal chelator of CQ protected against the neurotoxicity of 1-methyl-4-phenyl-1,2,3,6-tetra-pyridine (MTPT) in mice (101), which was biologically transformed to MPP+ by oxidation and induced that was parkinsonism clinically like PD (102). However, CQ is cytotoxic as a transition metal ionophore of such as Cu, Fe, and/or Zn and induces mitochondrial swelling and loss of mitochondrial membrane potential (103), acting as a potential anticancer agent (104). Moreover, CQ inhibits superoxide dismutase-1 (SOD-1) activity and enhances reactive oxygen (ROS) production, eventually leading to apoptosis in neuronal cells (105). Hence, the clinical efficacy of both CQ and PBT-2 therapies has also remained a matter of controversy due to incomplete understanding of the underlying physicochemical mechanism of oxidative stress in neurodegenerative diseases.

### Future in Application of Synchrotron Microbeam Techniques

From a physicochemical point of view, this paper presents a history of genetic epidemiological studies of ALS/PDC on Guam and the Kii Peninsula, which is focused on a role of environmental metallic elements, such as Ca, Mg, Al, Fe, Cu, and Zn, in the oxidative process of neuronal degeneration. Therefore, it is very important to regulate ionic homeostasis for therapeutic strategy to prevent the development and progression of neurodegenerative diseases (106).

A rapid development of synchrotron radiation (SR)-based studies in the recent decades provides us non-destructive analyses, chemical state analyses, and imaging distribution of the elements at a single cellular level (sensitivities in < 10^−6^ g [mg/Kg] and spatial resolution 2–10 μm) (107). For the therapeutic strategies, it is very important to elucidate a role of transition metals in the physicochemical mechanisms of oxidative stress on neurons, leading to the conformational changes in disease-related proteins, such as α-synuclein (Parkinson's disease, PD), amyloid β-peptide (Alzheimer's disease, AD), TDP-43 (amyotrophic lateral sclerosis/frontotemporal lobar degeneration, ALS/FTLD and frontotemporal lobar degeneration, FTLD), and so on (108). Recently, Fourier transformed infrared spectroscopy (FTIR) using SR microbeam has been applied to analyze secondary structure of β-amyloid deposits in Alzheimer's disease (109) and the intracytoplasmic β-sheet-rich structures of Lewy bodies in Parkinson's disease (110, 111). FTIR provides chemical information of tissue components such as proteins, lipids, nucleic acids, and carbohydrates and very sensitive to protein secondary structure, such as α-helical, β-sheet, and extended coil proteins (109–112). In near future, the combination of SXRF and FTIRM analyses will be a powerful tool to examine the misfolding of disease-specific proteins and accumulation of transition metals in various neurodegenerative diseases (113).

The application and development of SR microbeams in the fields of medicine and biology is expected to bring a new stage of future biomedical research and therapeutic strategies of ALS/PDC and other neurodegenerative diseases.

## Conclusion: Therapeutic Strategy and Prevention

Before World War II in Guam, the Chamorros used rainwater from tin roofs and ground surface water as a drinking water, containing extremely low contents of Ca and Mg. During 1970s, some 60 deep wells were begun to drill through the limestone formation to the water lens in the ground (average hardness of 250–300 ppm CaCO_3_). However, until 1977, there was not enough water supply for the south (less than 25 ppm CaCO_3_), where the incidence of ALS/PDC had been the highest (114). In the 1950s, the incidence of ALS and PDC on the island of Guam was much higher than those observed in the continental USA (115). From the late 1960s to the early 1980s, the incidence of both diseases has declined to the rates only slightly higher than that of elsewhere in the world (116). Concurrently, the leading cause of adult deaths in Guam has shift from ALS/PDC to cardiovascular and cerebral diseases and complications from type II diabetes, reaching the disease patterns of Westernized society (117).

On the other hand, after 1980s, the clustering of ALS/PDC in the Kii Peninsula has gradually disappeared by westernization of the lifestyle and socioeconomic changes, as well as Guam (118, 119). Recent environmental studies in the Kozagawa focus in the southernmost part of the Kii Peninsula of Japan have revealed; 1) high levels of Mn and Al in the soils, 2) markedly low levels of Ca, Mg, and Zn in the drinking water, 3) lower Ca and Zn levels in serum and higher urinary levels of 8-hydroxy-2'-deoxyguanosine (8-OHdG, an oxidative stress maker of DNA), and 3) high Cu/Zn ratio (a marker of oxidative stress) and intact PTH in serum of the patients with ALS and also some residents (119, 120). Furthermore, the spinal cord tissues and scalp hair of Kii ALS contained a high level of transition metals, i.e., Mn, V, and/or Ti (58, 121).

In 1975, the water source in Oshima (population: 1,217 in 2009, 9.9 Km^2^), a small island opposite to the top of the mainland of the Kii Peninsula, was changed from the Kozagawa river, in which the Ca and Mg contents were extremely low. Before 1975, the Oshima residents had taken a drinking water from wells and small rivers, rich in Ca and Mg and other minerals. Fujita et al. (122) compared the Ca metabolism of Oshima residents with that of Kozagawa residents. The Kozagawa residents in the mountainous area had thinner bone cortex and a higher frequency of lumbar spondylosis. The Oshima residents were mainly engaged in fishing and probably consumed abundant Ca from fish and shellfish. In Oshima, ALS had not been observed until 1999. However, three ALS cases appeared during the cross-sectional study from 2000 to 2009, which indicates that a long-term low intake of Ca and Mg over 25 years. It may play an important role in developing ALS among the Oshima residents (123).

Together with the results of extensive genetic environmental studies of both Kii and Guam ALS/PDC, it suggested that the lifestyle changes due to westernization in the recent years might exert a protective effect against the oxidative stress, finally leading to the decline or disappearance of ALS/PDC from both foci. On the public health prevention, it is important to correct environmental factors for protecting against the oxidative stress; 1) correcting low Ca and high Mn levels in drinking water and daily food, 2) improving the Cu/Zn ratio in serum, and 3) avoiding too much intake foods rich in transition metals, i.e., Al, V, and Ti, such as dried whole sardines, horse mackerels, and so on.

Currently, the only approved drugs for ALS are riluzole (glutamate antagonist) taken orally and edaravone (free radical scavenger) given intravenously. However, none of these drugs can ameliorate the progression of the disease (124). The importance of free radicals in the oxidative process of both sALS and fALS is an issue to be elucidate near future.

Globally and domestically, medical applications of SR-radiation microbeam are still very few, and their powerful analytical capabilities are not fully recognized. This technique has the advantage that they can be applied to biological specimens, such as autopsy cases, tissue specimens such as transgenic mice, cultured cells, and iPS cells in a living state without any damage in the air. By combining both XRF-XAFS and FTIR methods, it will provide a useful technique for translational interdisciplinary researches of drug discovery, using an iPS cell model of ALS in future (125).

## Author Contributions

The author confirms being the sole contributor of this work and has approved it for publication.

## Conflict of Interest

The author declares that the research was conducted in the absence of any commercial or financial relationships that could be construed as a potential conflict of interest.

## Publisher's Note

All claims expressed in this article are solely those of the authors and do not necessarily represent those of their affiliated organizations, or those of the publisher, the editors and the reviewers. Any product that may be evaluated in this article, or claim that may be made by its manufacturer, is not guaranteed or endorsed by the publisher.

## References

[1] Espinosa RE Okihiro MM Mulder DW Sayre GP. Hereditary amyotrophic lateral sclerosis. A clinical and pathological report with comments on classification. Neurol. (1962) 12:1–7. 10.1212/WNL.12.1.1

[2] Hirano A Kurland LT Krooth RS Lassell S. Parkinsonism-dementia complex, an endemic disease on the island of Guam. I. Clinical features. Brain. (1961) 84:642–61. 10.1093/brain/84.4.642 13907609

[3] Hirano A Malamud N Kurland LT. Parkinsonism-dementia complex, an endemic disease on the island of Guam. II. Pathological features. Brain. (1961) 84:662–79. 10.1093/brain/84.4.662 13907610

[4] Shiraki H Yase Y. Amyotrophic lateral sclerosis in Japan. In: de Jong MBV, editor. Handbook of Clinical Neurology, Vol. 22: System Disorders and Atrophies (Amsterdam; Oxford; New York, NY: North-Holland Pub; American Elsevier Pub) (1975). p. 354–419.

[5] Gajdusek DC Salazar AM. Amyotrophic lateral sclerosis and parkinsonian syndromes in high incidence among the Auyu and Jakai people of West New Guinea. Neurol. (1982) 32:107–26. 10.1212/WNL.32.2.107 7198738

[6] Jesus de Pedro-Cuesta Letivan I. Epidemiology of motor neuron disease (Chapter12), In: Anderson DW, Schoenberg DG, editors. Neuroepidemiology: A tribute to Bruce Schoenberg, Boston: CRC Press Inc. (1991). p. 265–96.

[7] Handa Y Yase Y. Genetico-epidemiological study concerning amyotrophic lateral sclerosis. Igaku no Ayumi. (1970) 73:478–84.

[8] Plato CC Garruto RM Fox KM Gajdusek DC. Amyotrophic lateral sclerosis and Parkinsonism-dementia on Guam: a 25-year prospective case study. Am J Epidem. (1986) 124:643–56. 10.1093/oxfordjournals.aje.a114437 3752057

[9] Brody JA Chen K-M. Changing epidemiologic patterns of amyotrophic lateral sclerosis and Parkinsonism-dementia on Guam. In: Norris, Kurland editors. Motor Neuron Diseases, Contemporary Neurology Symposia, Vol. II. New York, NY: Grune & Stratton (1969). p. 61–9.

[10] Reed DM Brody JA. ALS and PDC of Guam 1945-1972. I. Descriptive epidemiology. Am J Epidem. (1975) 101:287–301. 10.1093/oxfordjournals.aje.a112097 1124759

[11] Kurland LT. An update on the epidemiologic and etiologic perspectives of the amyotrophic lateral sclerosis/Parkinsonism-dementia complex in the Western Pacific. Neurol Forum. (1993) 3:3–14.

[12] Spencer PS Palmer VS Herman A Asmedi A. Cycad use and motor neuron disease in Irian Jaya. Lancet. (1987) 2:1273–4. 10.1016/S0140-6736(87)91883-6 2890883

[13] Spencer PS Ohta M Palmer VS. Cycad use and motor neuron disease in kii peninsula of Japan. Lancet. (1987) 2:1462–3. 10.1016/S0140-6736(87)91159-7 2892020

[14] Spencer PS Kisby GE Ludolph AC. Slow toxins, biologic marker, and long-latency neurodegenerative disease in the western Pacific region. Neurology. (1991) 41:62–6. 10.1212/WNL.41.5_Suppl_2.62 2041595

[15] Iwami O Watanabe T Moon CS Nakatuka H Ikeda M. Motor neuron disease on the Kii Peninsula of Japan: excess manganese intake from food coupled with low magnesium in drinking water as a risk factor. Sci Total Environ. (1994) 149:121–35. 10.1016/0048-9697(94)90010-8 8029710

[16] Kokubo Y Banaback SA Morimoto S Murayama S Togashi T Metcalf JS. β-N-methylamino-alanine analysis in the brains of patients with Kii ALS/PDC. Neurology. (2017) 89:1091–2. 10.1212/WNL.0000000000004310 28794246

[17] Spencer PS Palmer VS Kihira T Yoshida S Reis J Yabushita M. Kampo medicine and Muro disease (Amyotrophic lateral sclerosis and parkinsonism dementia complex): postscript and Historical Footnote. eNeurologicalSci. (2020) 22:100308. 10.1016/j.ensci.2020.100308 33426315 PMC7782320

[18] Spencer PS. Hypothesis: etiologic and molecular mechanistic leads for sporadic neurodegenerative diseases based on experience with Wester Pacific ALS/PDC. Front Neurol. (2019) 10:754. 10.3389/fneur.2019.00754 31417480 PMC6685391

[19] Yoshimasu F Yasui M Yase Y Iwata S Gajdusek DC Gibbs CJ Jr. Studies on amyotrophic lateral sclerosis by neutron activation analysis-2. Comparative study of analytical results on Guam PD, Japanese ALS and Alzheimer disease cases. Folia Psychiat Neurol Jpn. (1980) 34:75–82. 10.1111/j.1440-1819.1980.tb01515.x 7390331

[20] Yoshida S Wakayama I Kihira T Sasajima K Yoshida K. Environmental minerals in Kii amyotrophic lateral sclerosis in Japan: A PIXE analysis featuring aluminum. Int J PIXE. (1996). 6:543–54. 10.1142/s129083596000600

[21] Yase Y. The pathogenesis of amyotrophic lateral sclerosis. Lancet. (1972) 2:292–6. 10.1016/S0140-6736(72)92903-0 4115029

[22] Yase Y. The basic process of amyotrophic lateral sclerosis as reflected in Kii Peninsula and Guam. In: Proceedings of the 11th World Congress of Neurology, Amsterdam, September 1–16. Amsterdam (1977). p. 413–27.

[23] Yoshida S Kihira T Kohmoto J Wakayama I Uebayashi Y Matsumoto N. Environmental and emigration factors in amyotrophic lateral sclerosis: dormant Kii Peninsula, Japan, focus? In: Nakano I, Hirano A, editors. Amyotrophic Lateral Sclerosis: Progress and Perspectives in Basic Research and Clinical Application, Proceedings of the 11th Tokyo Metropolitan Institute for 25-27 October. Tokyo (1995). p. 158–62.

[24] Tsunoda K Yamashita T Shimada H Nomura E Takahashi Y Shang J et al. A migration case of Kii amyotrophic lateral sclerosis/parkinsonism dementia complex with the shortest stay in the endemic area and the longest incubation to develop the disease. J Clin Neurosci. (2018) 46:64–7. 10.1016/j.jocn.2017.08.057 28890043

[25] Torres JBS Iriarte LLG Kurland LT. Amyotrophic lateral sclerosis among Guamanians in California. Calif Med. (1957) 86:385–8. 13426814 PMC1511959

[26] Garruto RM Gajdusek DC Chen K-M. Amyotrophic lateral sclerosis and parkinsonism-dementia among Filipino migrants to Guam. Ann Neurol. (1981) 10:341–50. 10.1002/ana.410100405 7316487

[27] Yoshida S Yano I Wakayama I Mitani K Yase Y. Morphometric analysis of neurodegenerative changes induced by low calcium-magnesium and excess aluminum intake. Biomed Res. (1990) 11:11–18. 10.2220/biomedres.11.11

[28] Kihira T. Morphological, morphometrical and metal analytical studies of oral aluminum neurotoxicity. Brain Nerve. (1987) 39:633–41. 3675927

[29] Kihira T Yoshida S Uebayashi Y Wakayama I Yase Y. Experimental model of motor neuron disease: oral aluminum neurotoxicity. Biomed Res. (1994) 15:27–36. 10.2220/biomedres.15.279513930

[30] Garruto RM Shankar SK Yanagihara R Salazar AM Amylx HL Gajdusek DC. Low calcium, high aluminum diet-induced motor neuron pathology in cynomolgus monkeys. Acta Neuropathol. (1989) 78:210–9. 10.1007/BF00688211 2750490

[31] Kihira T Yoshida S Kondo T Yase Y Ono S Kondo T. Chronic low-Ca/Mg high-Al diet induces neuronal loss. Neuropathology. (2002) 22:171–9. 10.1046/j.1440-1789.2002.00441.x 12416556

[32] Mitani K. Relationship between neurological disease and aluminum load, especially amyotrophic lateral sclerosis. Magnesium Res. (1992) 5:203–13. 1467159

[33] Oyanagi K Kawakami E Kikuchi-Horie K Ohara K Ogata K Takahama S. Magnesium deficiency over generations in rats with special references to the pathogenesis of the Parkinsonism-dementia complex and amyotrophic lateral sclerosis of Guam. Neuropathology. (2006) 26:115–28. 10.1111/j.1440-1789.2006.00672.x 16708544

[34] Kolzlowski MA Williams C Hilton DR Williams C. Heterotopic neurons in spinal cord of patients with ALS. Neurology. (1989) 39:644–8. 10.1212/WNL.39.5.644 2710354

[35] Aoki M Ogasawara M Matsubara Y Narisawa S Nakamura S Ioyama Y. Mild ALS in Japan associated with novel SOD mutation. Nat Genet. (1993) 5:323–4. 10.1038/ng1293-323 8298637

[36] Rosen DR Siddique T Patterson D Figelwicz DA Sapp P Hentani A. Mutations in Cu/Zn superoxide dismutase gene are associated with familial amyotrophic lateral sclerosis. Nature. (1993) 362:59–62. 10.1038/364362c0 8446170

[37] Kabashi E Valdmanis PN Dion P Spiegelman D McCookey BIJ Velde CV et al. TARDBP mutations in individuals with sporadic and familial amyotrophic lateral sclerosis. Nat Genet. (2008) 40:572–4. 10.1038/ng.132 18372902

[38] Sreedharan J Blair IP Tripathi VB Hu X Vance C Rogelj B. TDP-43 mutations in familial and sporadic amyotrophic lateral sclerosis. Science. (2008) 319:1668–72. 10.1126/science.1154584 18309045 PMC7116650

[39] Kwiatkowski Jr TJ Bosco DA Lederc AL Tamrazian E Vanderburg CR Russ C. Mutations in the FUS/TLS gene on chromosome 16 cause familial amyotrophic lateral sclerosis. Science. (2009) 323:1205–8. 10.1126/science.1166066 19251627

[40] Vance C Rogelj B Hortobagyi T De Vos KJ Nishimura AL Sreedharan J et al. Mutations in FUS, an RNA processing protein, cause familial amyotrophic lateral sclerosis type 6. Science. (2009) 323:1208–11. 10.1126/science.1165942 19251628 PMC4516382

[41] DeJesus -Hermandez M Mackkenzie IR Boeve BF Boxer AL Baker M Ratherford NJ et al. Expanded GGGGCC hexanucleotide repeat in noncoding region of C9ORF72 causes chromosome 9p-linked FTD and ALS. Neuron. (2011) 72:245–56. 10.1016/j.neuron.2011.09.011 21944778 PMC3202986

[42] Renton AE Majounie E Waite A Simon-Sanchez J Rollinson S Gibbs JR et al. A Hexanucleotide repeat expansion in C9ORF72 is the cause of chromosome 9p21-linked ALS-FTD. Neuron. (2011) 72:257–68. 10.1016/j.neuron.2011.09.010 21944779 PMC3200438

[43] Gurney ME Pu H Chi AY Dal Canto MC Polchow CY Alexander DD et al. Motor neuron degeneration in mice that express a human Cu, Zn superoxide dismutase mutation. Science. (1994) 264:1772–5. 10.1126/science.8209258 8209258

[44] Reaume AG Elliot JL Hoffmann EK Kowall NW Ferrante RJ Siwek DF et al. Motor neurons in Cu/Zn superoxide dismutase-deficient mice develop normally but exhibit enhanced cell death after axonal injury. Nature Genet. (1996) 13:43–7. 10.1038/ng0596-43 8673102

[45] Taylor JP Brown Jr RH Cleveland DW. Decoding ALS: from genes to mechanism. Nature. (2016) 539:197–206. 10.1038/nature20413 27830784 PMC5585017

[46] Cook C Petrucelli L. Genetic convergence brings clarity to the enigmatic red line in ALS. Neuron. (2019) 101:1057–69. 10.1016/j.neuron.2019.02.032 30897357

[47] Naruse H Takahashi Y Kihira T Yoshida S Kokubo Y Kuzuhara S et al. Mutational analysis of familial and sporadic amyotrophic lateral sclerosis with OPTN mutation. Amyotrophic Lateral Sclerosis. (2012) 13:562–6. 10.3109/17482968.2012.684213 22708870

[48] Ishiura H Takahashi Y Mitsui J Yoshida S Kihira T Kokubo Y et al. C9ORF72 repeat expansion in amyotrophic lateral sclerosis in the Kii Peninsula of Japan. Arch Neurol. (2012) 69:1154–8. 10.1001/archneurol.2012.1219 22637429

[49] Nishiyama A Niihori T Warita H Izumi R Akiyama T Kato M et al. Comprehensive targeted next-generation sequencing in Japanese familial amyotrophic lateral sclerosis. Neurobiol Aging. (2017) 53:194.e1–194.e8. 10.1016/j.neurobiolaging.2017.01.004 28160950

[50] Yoshimasu F Tanaka S Hayashi T Iwata K Oka H. Familial motor neuron disease: autopsy findings in one of two brothers. Rinsho Shinkeigaku. (1977) 17:439–45. 912996

[51] Kikukawa K Nakano R Inuzuka T Kokubo Y Narita Y Kuzuhara S. A missense mutation in the SOD1 gene in patients with amyotrophic lateral sclerosis from the Kii Peninsula and its vicinity, Japan. Neurogenetics. (1997) 1:113–5. 10.1007/s100480050016 10732812

[52] Kokubo Y Kuzuhara S Narita Y Kikukawa K Nakano R Inuzuka T et al. Accumulation of neurofilaments and SOD1-immunoreactive products in a patient with familial amyotrophic lateral sclerosis with 113T SOD1 mutation. Arch Neurol. (1999) 56:1506–8. 10.1001/archneur.56.12.1506 10593307

[53] Rouleau GA Clark AW Rook K Pramatarova A Krizus A Suchowersky O et al. SOD1 mutation is associated with accumulation of neurofilaments in amyotrophic lateral sclerosis. Ann Neurol. (1996) 39:128–31. 10.1002/ana.410390119 8572658

[54] Orrell RW King AW Hilton DA Campbell MJ Lane RJ de Belleroche JS. Familial amyotrophic lateral sclerosis with a point mutation of SOD-1: intrafamilial heterogeneity of disease duration associated with neurofibrillary tangles. J Neurol Neurosurg Psychiatry. (1995) 59:266–70. 10.1136/jnnp.59.3.266 7673954 PMC486027

[55] Yoshida S. X-ray microanalytic studies on amyotrophic lateral sclerosis. I. Metal distribution compared with neuropathological findings in cervical spinal cord. Rinsho Shinkeigaku. (1977) 17:299–309. 560276

[56] Yoshida S. X-ray microanalytical studies on amyotrophic lateral sclerosis. II. The interrelationships of intraspinal blood supply metal deposition and degenerative changes. Rinsho Shinkeigaku. (1979) 19:283–91. 477130

[57] Yoshida S. X-ray microanalytic studies on amyotrophic lateral sclerosis. III. Relationship of calcification degeneration found in cervical spinal cord of ALS. Rinsho Shinkeigaku. (1979) 19:641–52. 544115

[58] Yoshida S Yase Y Iwata S Mizumoto Y Chen KM Gajdusek DC. Comparative trace-elemental study on amyotrophic lateral sclerosis (ALS) and parkinsonism-dementia complex in the Kii Peninsula of Japan and Guam. Wakayama Med Rep. (1988) 30:41–53.

[59] Iwata S. Structural analysis of metal coprecipitated calcification products in the central nervous system with particular reference to ALS. Neuro Med. (1980) 13:103–7.

[60] Yase Y. ALS in Kii Peninsula: one possible etiological hypothesis. In: Tubaki T, Toyokura Y. editors. ALS. Tokyo and Baltimore. Tokyo; Baltimore, MD : University of Park Press (1979). p. 307–318.

[61] Selye H. Topical calciphylaxis. in Calciphylaxis, Chicago: University of Chicago Press (1962). p. 48–54.

[62] Yoshida S Kihira T Mitani K Wakayama I Yase Y Yoshida H. Intraneuronal localization of aluminum: possible interaction with nucleic acids and pathogenetic role inamyotrophic lateral sclerosis ALS. In: Clifford Rose F, Norris F, editors. New Advances in Toxicology and Epidemiology. London: Smith-Gordon (1990) p. 211–23.

[63] Yoshida S Mitani K Kihira T Yase Y. Bunina body formation in amyotrophic lateral sclerosis: a morphometric-statistical and trace element study featuring aluminum. J Neurol Sci. (1995) 130:88–94. 10.1016/0022-510X(95)00011-P 7650536

[64] Kawahara M Kato-Negishi M. Link between aluminum and the pahogenesis of Alzheimer's disease: the integration of the aluminum and amyloid cascade hypotheses. Int J Alzheimers Dis. (2011) 2011:276393–410. 10.4061/2011/276393 21423554 PMC3056430

[65] Yoshida S. Environmental factors in Western Pacific Foci of ALS and a possible pathogenetic role of aluminum (Al) in motor neuron degeneration. Rinsho Shinkeigaku. (1991) 31:1310–2. 1817796

[66] Yoshida S Ari-Ide E. Application of a synchrotron radiation microbeam: Elemental and Chemical state analyses at cellular level in Parkinson's disease and amyotrophic lateral sclerosis. Biomed Res Trace Elements. (2003) 14:196–203.

[67] Yoshida S. Application of synchrotron radiation micro beam to medical research – Oxidative stress induced by transition metals on neurodegeneration of sporadic and familial amyotrophic lateral sclerosis. Biomed Res Trace Elements. (2017) 28:145–53.

[68] Yoshida S Ektessabi AI Fujisawa S. Application of synchrotron radiation in neuromicrobiology: role of Iron in Parkinson's disease. Struct Chem. (2003) 14:85–95. 10.1023/A:1021673127598

[69] Acone I Castaner R Tarricone C Bolognesi M Stropolo ME Desideri A. Evidence of His61 imidazolate bridge rupture induced crystalline Cu, Zn superoxide dismutase. Bioch Biophys Res Comm. (1997) 241:119–21. 10.1006/bbrc.1997.7777 9405243

[70] Ide-Ektessabi A Fujisawa S Yoshida S. Chemical state imaging of iron in nerve cells from a patient with parkinsonism-dementia complex. J Appl Phys. (2002) 91:1613–7. 10.1063/1.1426244

[71] Crow JP Sampson JB Ahuang Y Thompson JA Beckman JS. Decreased zinc affinity of amyotrophic lateral sclerosis-associated superoxide dismutase mutants lead to enhanced catalysis of tyrosine nitration by peroxynitrite. J Neurochem. (1977) 69:1936–44. 10.1046/j.1471-4159.1997.69051936.x 9349538

[72] Pierson KB Evenson MA. 200 kd neurofilament protein binds Al, Cu and Zn. Biochem Biophys Res Commum. (1988) 152:598–604. 10.1016/S0006-291X(88)80080-9 3130052

[73] Bahar E Kim H Yoon H. ER stress-mediated signaling: action potential and Ca2+ as key players. Int. J. Mol. Sci. (2016) 17:1558–79. 10.3390/ijms17091558 27649160 PMC5037829

[74] Castilho RF Carvalho-Alves PC Vercesi AE Ferreira ST. Oxidative damage to sarcoplasmic reticulum Ca (2+)-pump induced by Fe^2+^/H_2_O_2_/ascorbate is not mediated by lipid peroxidation or thiol oxidation and leads to protein fragmentation. Mol Cell Biochem. (1996) 59:105–14. 10.1007/BF00420912 8858560

[75] Okumura H Chen KM Kurland LT. Recent epidemiological study of ALS/PDC in Guam Island. Jap Clin Epidemiol. (1995) 4:24–28.

[76] Chen KM. Disappearance of ALS from Guam: implications for exogenous causes. Clin Neurol. (1995) 35:1549–53. 8752460

[77] Hirano A Zimmerman HM. Alzheimer's neurofibrillary changes: a topographic study. Arch Neurol. (1962) 7:227–42. 10.1001/archneur.1962.04210030065009 13907611

[78] Yoshida S Ektessabi A Kitamura N Shikine S Fujisawa S Wakayama I. Iron and oxidative stress in nigral neurons of Guamanian ALS/PDC: Chemical state imaging using synchrotron radiation. In: Abe K, editor. Molecular Mechanism and Therapeutics of Amyotrophic Lateral Sclerosi. Amsterdam: Elsevier Science B.V. (2001). p. 227–33.

[79] Krop AJ Bunker BA Eiser M Moss SC Zecca L Stroppolo A. X-ray absorption fine-structure spectroscopy studies of Fe sites in natural human neuromelanin and synthetic analogue. Biophys J. (1990) 2:3135–42. 10.1016/S0006-3495(98)77755-0 9826634 PMC1299985

[80] Jellinger K Paulus W Riederer P Toudim MBH. Brain iron and ferritin in Parkinson's and Alzheimer's diseases. J Neural Transm. (1990) 2:327–40. 10.1007/BF02252926 2078310

[81] Halliwell B. Reactive oxygen species and the central nervous system. J Neurochem. (1992) 59:1609–23. 10.1111/j.1471-4159.1992.tb10990.x 1402908

[82] Weiss J. Reaction mechanism of oxidation-reduction process. Nature. (1934) 133:648–9. 10.1038/133648c0

[83] Enami S Sakamoto Y Coluss AJ. Fenton chemistry at aqueous interfaces. Proc Natl Acad Sci USA. (2014) 11:623–8. 10.1073/pnas.1314885111 24379389 PMC3896178

[84] Ikawa M Okazawa H Tsujikawa T Matsunaga A Yamamura O Mori T et al. Increased oxidative stress in related to disease severity in the ALS motor cortex: PET study. Neurology. (2015) 84:2033–9. 10.1212/WNL.0000000000001588 25904686

[85] Neuman M Sampathu DM Kwong LK Traux AC Micsenyi MC Chou TT et al. Ubiquitinated TDP-in frontotemporal lobar degeneration and amyotrophic lateral sclerosis. Science. (2006) 314:130–3. 10.1126/science.1134108 17023659

[86] Mori F Kakita A Takahashi H Wakabayashi K. Co-localization of Bunina bodies and TDP-43 inclusions in lower motor neurons in amyotrophic lateral sclerosis. Neuropathology. (2014) 34:71–6. 10.1111/neup.12044 23711197

[87] Miki Y Mori F Seino Y Tanji K Yoshizawa T Kijima H. Colocalization of Bunia bodies and TDP-43 inclusions in a case of sporadic amyotrophic lateral sclerosis. Neuropaholgy. (2018) 38:521–8. 10.1111/neup.12484 29938835

[88] Niebroj-Dobosz I Dziewulska D Kwiecinski H. Oxidative damage to proteins in the spinal cord in amyotrophic lateral sclerosis (ALS). Folia Neuropahol. (2004) 42:151–6. 15535033

[89] Dang TNT Lim NHK Grubman A Li Q-X Volitakis I White AR. Increased metal content in the TDP-43A315T transgenic mouse model of frontotemporal lobar degeneration and amyotrophic lateral sclerosis. Front Aging Neurosci. (2014) 6:15. 10.3389/fnagi.2014.00015 24575040 PMC3920072

[90] Lopez-Gonzalez R Lu Y Gendron TF Karydas A Tran H Yang D et al. Poly(GR) in C9orf71-Related ALS/PDC compromises mitochondrial function and increases oxidative stress and damage in iOSC-drived motor neurons. Neuron. (2016) 92:383–91. 10.1016/j.neuron.2016.09.015 27720481 PMC5111366

[91] Konno T Shiga A Tsujino A Sugai A Kato T Kanai K et al. Japanese amyotrophic lateral sclerosis patients with GGGGCC hexanucleotide repeat expansion in C9ORF72. J Neurol Neurosurg Psychiatry. (2013) 84:398–401. 10.1136/jnnp-2012-302272 23012445

[92] Doraiswamy PM Finefrock AE. Metals in our minds: therapeutic implications for neurodegenerative disorders. Lancet Neurol. (2004) 3:431–4. 10.1016/S1474-4422(04)00809-9 15207800

[93] Park MH Lee SJ Byun HR Kim Y Oho YJ KohJY et al. Clioquinol induces autophagy in cultured astrocytes and neurons by acting as a zinc ionophore. Neurobiol Dis. (2011) 42:242–51. 10.1016/j.nbd.2011.01.009 21220021

[94] Treiber C Simons A Strauss M Hafner M Cappai R Bayer TA et al. Clioquinol mediates copper uptake and counteracts copper efflux activities of the amyloid precursor protein of Alzheimer's disease. J Biol Chem. (2004) 279:51958–64. 10.1074/jbc.M407410200 15465814

[95] Cherny RA Atwood CS Xilians ME Gray DN Jones WD McLean CA et al. Treatment with a copper-zinc chelator markedly and rapidly inhibits β-amyloid accumulation in Alzheimer's disease transgenic mice. Neuron. (2001) 30:665–76. 10.1016/S0896-6273(01)00317-8 11430801

[96] Ritchie GW Bush AI Mackinnon A Maccfarlane S Mastwsky M MacGregor L et al. Metal-protein attenuation with iodochlorhydroxyquin (clioquinol) targeting A beta amyloid deposition and toxicity in Alzheimer disease: a pilot phase 2 clinical trial. Arch Neurol. (2003) 60:1685–91. 10.1001/archneur.60.12.1685 14676042

[97] Tabira T. Clioquinol's return: caution from Japan. Science. (2001) 292:2251–2. 10.1126/science.292.5525.2251 11424945

[98] Lannfelt L Blennow Kaj Zetterburg H Batsman S Ames D Harrison J et al. Safety, efficacy, and biomarker findings of PBT2 in targeting A beta as a modifying therapy for Alzheimer's disease: a phase IIa, double-blind, randomized, placebo-controlled trial. Lancet Neurol. (2008) 7:779–86. 10.1016/S1474-4422(08)70167-4 18672400

[99] Faux NG Richie CW Gunn A Rembach A Tsatsanis A Bedo J et al. PBT2 rapidly improves cognition in Alzheimer's disease: additional phase II analyses. J Alzheimers Dis. (2010) 20:509–16. 10.3233/JAD-2010-1390 20164561

[100] Schäfer S Pajonk FG Multhaup G Bayer TA. Copper and clioquinol treatment in young APP transgenic and wild-type mice effects on life expectancy, body weight, and metal-ion levels. J Mol Med (BERL). (2007) 85:405–13. 10.1007/s00109-006-0140-7 17211610

[101] Kaur D Yantiri F Rajagopalan S Kumar J Mo JQ Boonplueang R et al. Genetic or pharmacological iron chelation prevents PTP-induced neurotoxicity in vivo: a noveltherapy for Parkinson's disease. Neuron. (2003) 37:899–909. 10.1016/S0896-6273(03)00126-0 12670420

[102] Burns RS LeWitt PA Ebert MH Pakkenberg H Kopin IJ. The clinical syndrome of striatal dopamine deficiency. Parkinsonism induced by 1-methyl-4-phenyl-1,2,3,6-tetrahydropyridine (MPTP). New Engl J Med. (1985) 312:1418–21. 10.1056/NEJM198505303122203 2581135

[103] Feng P Li TI Guan ZX Franklin RB Costello LC. Direct effect of zinc on mitochondrial apoptogenesis in prostate cells. Prostate. (2002) 52:311–8. 10.1002/pros.10128 12210492 PMC4465826

[104] Ding W-Q Liu B Vaught JL Yamauchi H Lind SE. Anticancer activity of the antibiotic clioquinol. Cancer Res. (2005) 65:3389–95. 10.1158/0008-5472.CAN-04-3577 15833873

[105] Kawamura K Kuroda Y Sogo M Fujimoto M Inui T Mitsui T. Superoxide dismutase as a target of clioquinol-induced neurotoxicity. Biochem Biophys Res Commun. (2014) 452:181–5. 10.1016/j.bbrc.2014.04.067 24755073

[106] Sirabella R Valsecchi V Anzilotti S Cuomo O Vinciguerra A Cepparulo P et al. Ionic homeostasis maintenance in therapeutic Targets. Front Neurosci. (2018) 12:510. 10.3389/fnins.2018.00510 30131665 PMC6090999

[107] Sutton SR Bertsch PM Newville M Revers M Ranziroti A Eng P. Microfluorescence and microtomography analyses of heterogenous earth and environmental materials. In: Fenter PA, Rivers ML, Sturchio NC, Sutton SR, editors. Applications of Synchrotron Radiation in Low-Temperature Geometry and Environmental Science, Vol. 49. Washington, DC: Reviews in Mineralogy & Geochemistry; Mineralogical Society of America (2002). p. 579.

[108] Dugger BN Dikson DW. Pathology of neurodegenerative diseases. Cold Spring Harb Prospect Biol. (2017) 9:a028035. 10.1101/cshperspect.a028035 28062563 PMC5495060

[109] Miller LM Wang Qi Telivara TP Smith RJ Lanzirotti A Miklossy J. Synchrotron-based infrared accumulation of Cu and Zn co-localized with β-amyloid deposits in Alzheimer's disease. J Structural Biol. (2006) 155:30–37. 10.1016/j.jsb.2005.09.004 16325427

[110] Araki K Yagi N Ikemoto Y Yagi H Choog CJ Hayakawa H et al. Synchrotron FTIR microspectroscopy for structural analysis of Lewy bodies in the brain of Parkinson's disease patients. Sci Rep. (2015) 5:17625. 10.1038/srep17625 26621077 PMC4664933

[111] Araki K Yagi N Ikemoto Y Hayakawa H Fujimura H Moriwaki T et al. The secondary structural difference between Lewy body and glial cytoplasmic inclusion in autopsy brain with synchrotron FTIR micro-spectroscopy. Sic Rep. (2020) 10:19423. 10.1038/s41598-020-76565-6 33173082 PMC7656264

[112] Byler DM Sui H. Examination of the secondary structure of proteins by deconvolved FTIR spectra. Biopolymers. (1986) 25:469–87. 10.1002/bip.360250307 3697478

[113] Sui H Byler DM Purcel JM. Estimation of beta-structure content proteins by means of deconvoluted FTIR spectra. J Biochem Biophys J. (1985) 11:235–40. 10.1016/0165-022X(85)90005-3 4067173

[114] Chen KM. Changing ecology and socioeconomy. In: Chen KM, editor. The Mysterious Diseases of Guam. Mangilao: Micronesian Area Research Center, University of Guam (2004). p. 221–3.

[115] Garruto RM Yanagihara R Gajdusek DC. Disappearance of high-incidence amyotrophic lateral sclerosis and parkinsonism-dementia complex on Guam. Neurology. (1985) 35:193–8. 10.1212/WNL.35.2.193 3969206

[116] Plato CC Garruto RM Galasko D Craig UK Plato M Gamst A et al. Amyotrophic lateral sclerosis and parkinsonism-dementia complex of Guam: changing incidence rates during the past 60 years. Amer J Epidemiol. (2003) 157:149–57. 10.1093/aje/kwf175 12522022

[117] Haddock R Chen KM. ALS and Diabetes on Giam: Changing patterns of chronic diseases in an island community. Southest Asian J Trop Med Pub Health. (2003) 34:221–3. 15115147

[118] Yoshida S Uebayashi U Kihira T Kohmoto J Wakayama I Taniguchi S et al. Epidemiology of motor neuron disease in the Kii Peninsula of Japan, 1989-1993: active focus or disappearing focus. J Neurol Sci. (1998) 155:146–55. 10.1016/S0022-510X(97)00300-6 9562259

[119] Kihira T Yoshida S Hironishi M Miwa H Okamoto K Kondo T. Changes in the incidence of amyotrophic lateral Sclerosis in Wakayama, Japan. Amyotrophic Lateral Scler Other Motor Neuron Disod. (2005) 6:155–63. 10.1080/14660820510030031 16183557

[120] Kihira T Okamoto K Yoshida S Kondo T Iwai K Wada S et al. Environmental characteristics and oxidative stress of inhabitants and patients with amyotrophic lateral sclerosis in a high-incidence area on the Kii Peninsula, Japan. Intern Med. (2013) 52:1479–86. 10.2169/internalmedicine.52.9521 23812195

[121] Kihira T Sakurai I Yoshida S Wkayama I Takamiya K Okumura R et al. Neutron activation analysis of scalp hair from ALS patients and residents in the Kii Peninsula, Japan. Biol Trace Elem Res. (2015) 164:36–42. 10.1007/s12011-014-0202-6 25524522

[122] Fujita T Okamoto Y Tomita T Ota K. Calcium metabolism in aging inhabitants of mountain versus seacoast communities in the Kii Peninsula. J Amer Geriat Soc. (1977) 25:254–8. 10.1111/j.1532-5415.1977.tb00410.x 864171

[123] Kihira T Yoshida S Kondo T Iwai K Wada S Morinaga S et al. An increase in ALS incidence on the Kii Peninsula, 1960-2009: a possible link to change in drinking water source. Amyotroph Lateral Scler. (2012) 13:347–50. 10.3109/17482968.2012.674140 22632441 PMC3409458

[124] Jaiswal MK. Riluzole and edaravone: a tale of two amyotrophic lateral sclerosis drugs. Med Res Rev. (2019) 39:733–48. 10.1002/med.21528 30101496

[125] Okano H Yasuda D Fujimori K Morimoto S Takahashi S. Ropinirole, a new ALS drug candidate developed using iPSCs. Trends Pharmacol Sci. (2019) 41:99–109. 10.1016/j.tips.2019.12.002 31926602

